# Challenges and Complication Management in Novel Artificial Iris Implantation

**DOI:** 10.1155/2018/3262068

**Published:** 2018-09-23

**Authors:** Christian S. Mayer, Andrea E. Laubichler, Ramin Khoramnia, Tamer Tandogan, Philipp Prahs, Daniel Zapp, Lukas Reznicek

**Affiliations:** ^1^Department of Ophthalmology, Technical University of Munich, Munich, Germany; ^2^Department of Ophthalmology, University of Heidelberg, Heidelberg, Germany; ^3^Department of Ophthalmology, University of Regensburg, Regensburg, Germany

## Abstract

**Purpose:**

Evaluation of postoperative artificial iris prosthesis-related complications.

**Design:**

Retrospective cohort study.

**Methods:**

Fifty-one consecutive patients underwent pupillary reconstruction using an artificial iris implant made from silicone between 2011 and 2015. Quantity and quality of complications were subclassified into three groups including mild, moderate, and severe complications. Their management and the learning curve were evaluated.

**Results:**

In total, 13 (25.5%) of 51 included artificial iris implantations showed unexpected events in various degrees: mild complications: recurrent bleeding (*n*=1, 2.0%), slight but stable iris deviation (*n*=1, 2.0%), capsular fibrosis (*n*=2, 3.9%); moderate complications: suture cutting through the residual iris (*n*=1, 2.0%), glaucoma (*n*=3, 5.9%), and corneal decompensation (*n*=3, 5.9%); severe complications: artificial iris suture loosening (*n*=2, 3.9%) and dislocation (*n*=3, 5.9%), synechiae (*n*=2, 3.9%), glaucoma (*n*=2, 3.9%), and corneal decompensation (*n*=5, 9.8%) with the need for surgery, cystoid macular edema (*n*=3, 5.9%) and retinal detachment (*n*=1, 2.0%). The complication rate decreased from 83.3% (5 of 6 implantations) in the first year to 13.3% (2 of 15 implantations) in the 4th year. Nineteen of 45 evaluated patients showed a significant gain in best-corrected visual acuity (BCVA) from 1.09 ± 0.56 logMAR to 0.54 ± 0.48 logMAR (*p* < 0.001), and 13 of 45 eyes had a significant BCVA loss from 0.48 ± 0.39 logMAR to 0.93 ± 0.41 logMAR after surgery (*p* < 0.001).

**Conclusions:**

The artificial iris is a feasible option in the treatment of iris defects with a wide spectrum of postoperative complications. The significant reduction of complications after twelve implantations implicates that the procedure is not to be recommended in low volume settings.

## 1. Introduction

Patients with iris defects suffer from severe visual impairment with especially increased glare sensitivity and cosmetic disturbances. In addition to those iris defects, these eyes show corneal and scleral scars, aphakia, retinal changes, and glaucoma depending on the initial trauma. Furthermore, iris atrophy and pupil size can deteriorate the visual function of an increase of higher order aberrations [1]. In the past, various types of artificial iris implants were used to address those iris defects [2–7]. Most of them were rigid, needed large incisions, and were not satisfying for the patients.

A custom-made, flexible silicone iris implant is a relatively new and promising additional option for the surgical treatment of iris defects [8–10]. The ArtificialIris® (HumanOptics) is a foldable, custom-tailored iris prosthesis made of flexible, biocompatible silicone. This device received the Conformité Européenne (CE) conformity marking for products sold within the European Economic Area approval for Europe in 2011 and received a Food and Drug Administration (FDA) approval procedure in the United States recently. Several surgeons have presented different cases or small case series of pupil and iris reconstruction using this novel type of silicone iris implant with good results [10–16]. In a previous publication, we were able to present good functional outcomes in pupillary reconstruction after artificial iris implantation in a larger series with 36 eyes [17]. The presented ArtificialIris must not be confused with the BrightOcular iris prosthesis (Stellar Devices). Numerous publications evaluating that device report concerns [18, 19] with only few good results [7].

There are still very few published studies addressing complications associated with the implant or the implantation procedure itself [13, 20, 21]. There is currently a need to investigate potential complications and pitfalls in this relatively new and rising therapeutic method in a larger amount of patients.

The purpose of this retrospective cohort study was to describe the learning curve of the implantation surgery, limitations, pitfalls, and associated unexpected events and their potential solutions for the use of this iris prosthesis.

## 2. Materials and Methods

Fifty-one silicone iris prostheses (ArtificialIris®, HumanOptics, Erlangen, Germany) were implanted at the eye clinic of the Technical University of Munich between June 2011 and December 2015 (Table 1).

The number and quality of complications associated with the implantation procedure or the implant itself were investigated for all included 51 patients with a follow-up time of at least 3 months to a maximum of 4 years.

Informed consent was obtained from all participants. The study was conducted according to the tenets of the Declaration of Helsinki, and approval by the Institutional Review Board was obtained.

The type of implantation procedure depended on the preexisting alterations in the affected eyes [22, 23]. In our cohort, all patients received either a complete (*n*=49) or a sector-shaped (*n*=2) iris prosthesis. The custom-made implant was fixed in the ciliary sulcus without sutures in case of a preexisting intracapsular intraocular lens, implanted in the capsular bag together with a new intraocular lens or sutured to the sclera with or without an attached intraocular lens. In two cases, an artificial iris segment was sutured directly in the sectoral iris defect. At the end of surgery, all patients were pseudophakic. Details of the implantation procedure are described elsewhere [17, 23]. All implantations were performed by one single surgeon without any notable intraoperative complications.

Postoperative changes, abnormalities, complications, and unexpected events associated with the artificial iris implantation were noted in standardized full ophthalmic examinations. The development of best-corrected visual acuity (BCVA, Snellen chart) was classified into three groups: BCVA improvement (>2 lines), unchanged BCVA (±2 lines), and decrease in BCVA (>2 lines). The unexpected events and complications during the follow-up period were grouped into “none” (postoperative uneventful course of disease), “mild” (unexpected events requiring noninvasive intervention *with* full recovery), “moderate” (unexpected events requiring noninvasive intervention *without* full recovery), and “severe” (unexpected or expected events resulting in any surgical intervention).

In addition, the complications were evaluated in regard to ocular hyper- and hypotension, suture fixation-associated problems, and misalignment and (sub-)luxation of the artificial iris.

Imaging documentation was performed with photography (Canon 600D SLR including a 100 mm Macro-Objective, Canon Europe LTD, United Kingdom UB11 1ET, and Topcon OneDigiPro 3HD Slit Lamp, Oakland, NJ 07436) and video capturing (Zeiss Lumera 700 ophthalmic microscope Callisto System, Carl Zeiss AG, Oberkochen, Germany).

## 3. Results

In this retrospective analysis, we were able to evaluate the complications of 51 patients, who had received an artificial iris implantation performed by one single surgeon in a university eye clinic setting between 2011 and 2015. Thirty-four (66.7%) were male and 17 (33.3%) were female. Mean age was 52.9 ± 16.0 years. The iris defects were due to congenital coloboma (*n*=3; 6.8%), persistent mydriasis (*n*=14; 27.4%), traumatic loss of iris tissue (*n*=31; 60.8%), and miscellaneous (*n*=3; 6.8%). Additional history information revealed preexisting glaucoma in 6 (11.8%) and preexisting corneal impairment (scars or decompensation) in 5 (9.8%) patients. The mean postoperative follow-up period is 13.4 months (min. 3 months and max. 50 months). The postoperative history includes a variety of individual complications ranging from none over moderate intraocular pressure rise for a few days up to a prosthesis explantation due to chronic inflammation and decompensation of the corneal endothelium. Thirty-eight procedures (74.5%) had an uneventful follow-up after artificial iris implantation.

The overall complication rate after surgery included 13 out of 51 patients (25.5%), and the complications were further subclassified into mild, moderate, and severe.

Mild complications manifested in recurrent bleeding into the anterior chamber and secondary rise of the intraocular pressure (IOP) (*n*=1, 2.0%) with spontaneous solution after 2 months, slight but stable artificial iris deviation (*n*=1, 2.0%), and capsular fibrosis (*n*=2, 3.9%) treated with laser-assisted capsulotomy.

**Figure 1:**
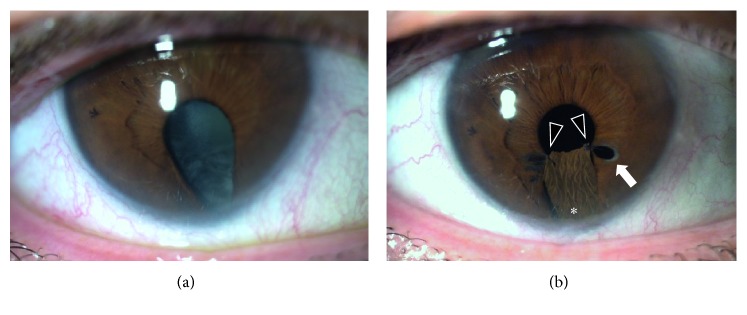
Congenital iris coloboma with cataract before (a) and after (b) implantation of a sector-shaped artificial iris (^*∗*^) and intraocular lens in a combined surgery. Three months after surgery, the suture at the pupillary rim (arrowheads) cut through the remnant iris tissue and formed a new pupillary aperture (arrow). This patient did not suffer from visual impairment.

Moderate complications were found in sutures cutting through the residual iris tissue (*n*=1, 2.0%) forming a secondary pupil without the need for intervention (Figure 1), onset of glaucoma (*n*=3, 5.9%) that could be controlled with topical antiglaucomatous medication, and corneal decompensation (*n*=3, 5.9%).

**Figure 2:**
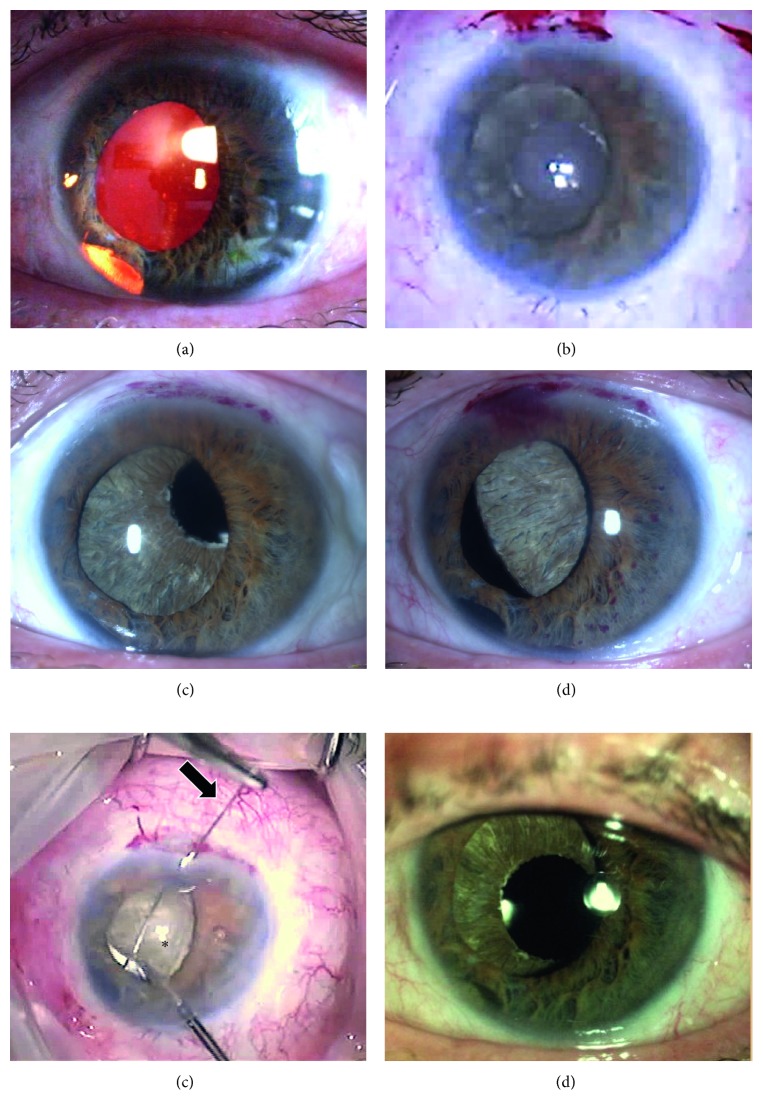
Patient (51 y, m) with an initially traumatic iris defect and a sclera-fixed intraocular lens (a). At the end of surgery, the artificial iris was located in the ciliary sulcus without sutures (b). (c) Slight decentralization of the pupillary aperture and subluxation of the artificial iris the first day after surgery. (d) Severe dislocation of the implant threatening to fall down into the vitreous the second day after surgery. Immediate operative intervention (e) with picking up the implant and fixing the device with a double-armed 10.0 polypropylene suture to the sclera (arrow). (f) Stable location of the implant and intraocular lens 1 year after surgery.

**Figure 3:**
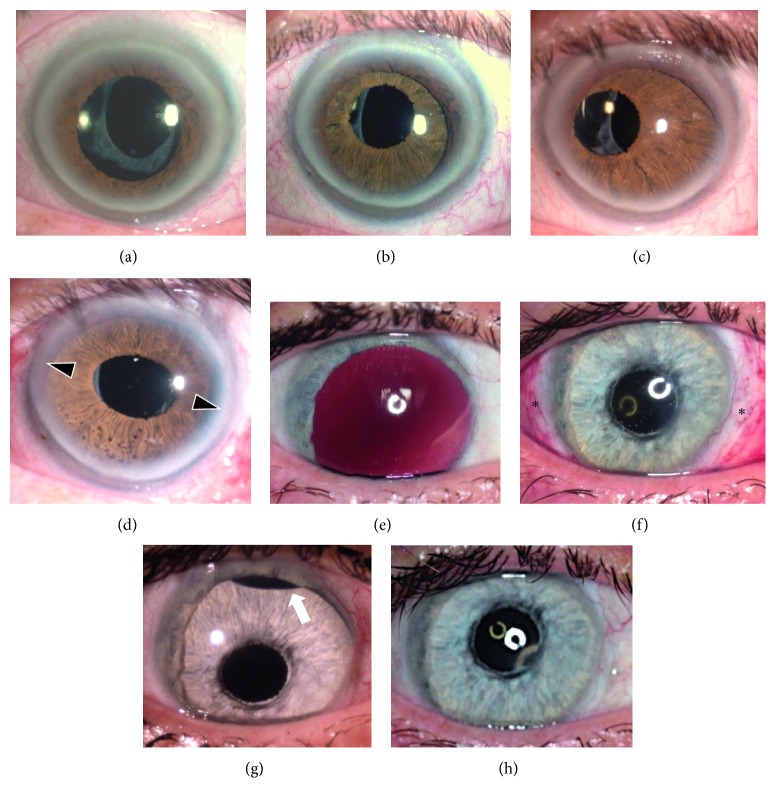
Two patients after artificial iris implantation with subluxation of the implant: Patient 1 (a–d) suffered from persistent traumatic mydriasis (a) and received a ciliary sulcus embedded fiber-free prosthesis without suture fixation (b). The prosthesis decentrated after 2 years with visual disturbances (c) and had to be refixed with sutures (d). This led to slight oval pupil shape. Patient 2 (e–h) suffered from aphakic and subtotal aniridia. An intraocular lens was attached to the artificial iris by sutures. This “sandwich” was implanted and sutured to the sclera at the 3 and 9 o'clock position (f, ∗∗). After 4 weeks, the implant tilted with the upper rim into the anterior chamber (g, arrow). Refixation with a third suture at the 11 o'clock position resolved that problem (h).

Severe complications were artificial iris suture loosening and dislocation with the necessity of surgical revision: Immediate postoperative subluxation of the artificial iris was detected in 3 cases (5.9%) of our collective (Figures 2 and 3).

All cases of significant misalignment and (sub)luxation of the artificial iris needed refixation of the implant. The subluxated irides were reattached with a u-type double-armed suture without significant tension to prevent a repeated cutting through (Figure 2(e); Supplementary Video, available here).

**Figure 4:**
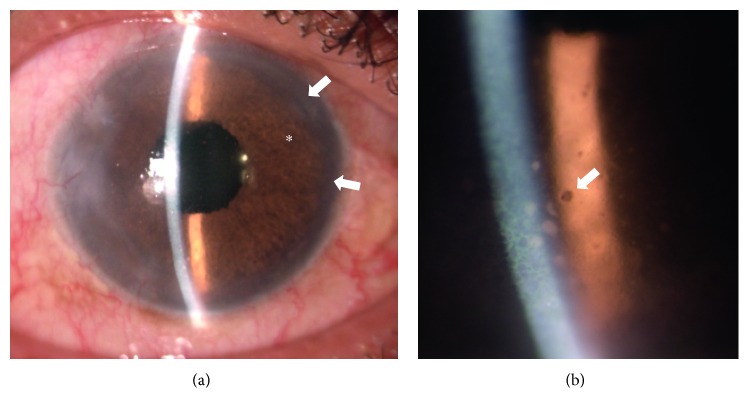
Anterior segment 2 years after artificial iris implantation with originally subtotal traumatic mydriasis (a) with intraocular pressure elevation (32 mm/Hg). Brown artificial iris (^*∗*^) with fixed pupillary aperture. Residual iris rim is barely visible (arrows). Magnification of the cornea (b): pigment dispersion and clumps in Arlt's triangle on the endothelium (arrow).

Formation of posterior synechiae requiring synechiolysis occurred in 3.9% (*n*=2). Severe ocular hypertension was observed in one case with severe pigment dispersion syndrome (Figures 4(a) and 4(b)) and in another case a new onset of glaucoma with the need for shunt surgery with Ahmed® glaucoma valves (*n*=2, 3.9%). Ocular hypotension after surgery was without exception transient for few days only.

**Figure 5:**
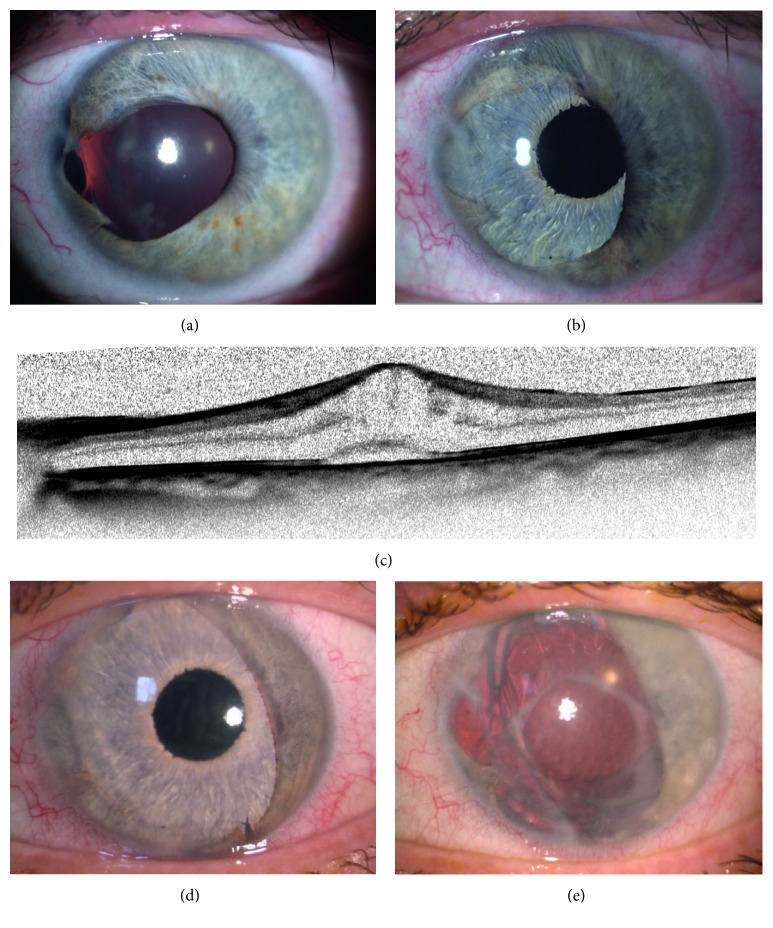
A 47-year-old woman with a 34-year iris defect and cataract after trauma (a). Results one year after artificial iris implantation: BCVA 20/20, clear media and stable situation (b). Two months later, the patient complained of a decreased BCVA of 20/200 and elevated IOP. Optical coherence tomography showed a cystoid macular edema (c), and slit lamp examination revealed descemet folds and corneal haze (d). Topical, intravitreal, and systemic medication did not improve BCVA. Explantation of the artificial iris (e) followed by perforating keratoplasty due to chronic inflammation and corneal decompensation. BCVA was 40/200 one year later.

Corneal complications occurred in 9.8% (*n*=5): Corneal decompensation resulted in perforating keratoplasty in 3 cases (5.9%) or amniotic membrane transplantation in 2 cases (3.9%). One of these cases developed an unexpected corneal decompensation one year after implantation surgery (Figure 5).

Initially, the BCVA in this case had recovered to 20/20 after the artificial iris implantation surgery. Furthermore, this patient developed a macular edema with corneal decompensation and consecutive vision decrease to 20/200 one year after the implant procedure. BCVA did not improve despite medical and surgical (amniotic membrane transplantation) treatment. Consecutively, the artificial iris had to be explanted one year after implantation.

A formation of cystoid macular edema (*n*=3, 5.9%) required intravitreal steroid application, and one case of retinal detachment (*n*=1, 2.0%) needed vitrectomy with silicone oil filling. The summary of the unexpected events is shown in Table 2.

In the first year (2011), we found 5 complications in 6 implantation procedures (83.3%), and in the second year (2012), we had 3 documented unexpected events in 7 surgeries (42.9%). In the following years, there were 3 complications in 12 procedures (25.0%, 2013), none in 11 (2014), and 2 in 15 procedures (13.3%, 2015).

**Figure 6:**
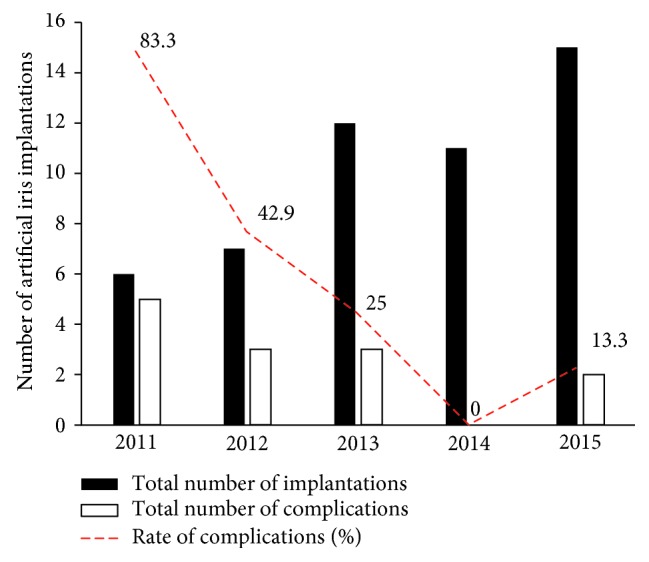
Incidence of complications after artificial iris implantation and complication rate. The surgeon's learning curve can be seen in the decreasing rate of complications with increasing numbers of implantations over the time. After 2 years—or 13 artificial iris implantations—the complication rate is below 25% and the surgical learning curve is significantly flattened.

Overall, 46.2% of all complications occurred within the first 3 postoperative months, whereas 53.8% occurred after that time period. The surgical learning curve with complications versus number of operations can be seen in Figure 6.

**Figure 7:**
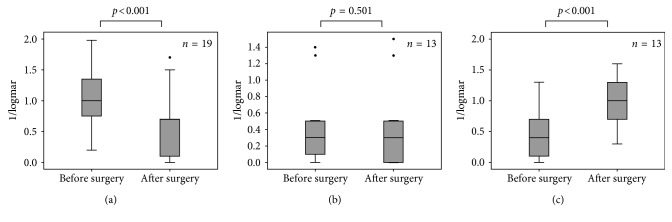
Development of visual acuity (logMAR) in 45 eyes after artificial iris implantation. Nineteen patients had a significant BCVA increase (>2 lines of a Snellen projection chart, *p* < 0.001) (a); 13 patients remained unchanged (±2 lines of a Snellen projection chart, *p*=0.501) (b); 13 patients (37.8%) suffered from a significant BCVA decrease (>2 lines of a Snellen projection chart, *p* < 0.001) (c).

We were able to analyze the BCVA development (preoperative versus postoperative) in 45 out of 51 patients. The BCVA increased significantly (more than 2 lines of a Snellen projection chart) in 19 patients (42.2%) from initially 1.09 ± 0.56 logMAR to 0.54 ± 0.48 logMAR after surgery (*p* < 0.001). In 13 eyes (28.9%), the BCVA remained unchanged (0.47 ± 0.54 logMAR to 0.45 ± 0.55 logMAR) after surgery (*p*=0.502), and in 13 eyes (28.9%), the BCVA decreased significantly from 0.48 ± 0.39 logMAR to 0.93 ± 0.41 logMAR after surgery (*p* < 0.001) (Figure 7).

## 4. Discussion

The initial complication rate after artificial iris implantation in the first year is very high (83.3%) due to case mix of rather complicated cases, an unfamiliar implant device, and relatively few and not standardized cases. We were able to show that, roughly after two years, the complication rate decreases indirectly proportional to the increasing experience and the learning effect of the surgeon (Figure 6). Our data suggest a dozen of artificial iris implantations be performed by one surgeon in order to achieve a certain level of standardization with an acceptable complication rate over time. Further investigations will show a possible increase of late-onset complications in eyes with currently short follow-up periods at present.

Best-corrected visual acuity (BCVA) improvement is not a primary goal for the implantation of an artificial iris. Affected eyes often have multiple alterations and a reduced BCVA prognosis. Increase of the BCVA after surgery in 19 patients (42.2%) may result from cataract extraction and/or IOL implantation or by reconstruction of a new pupil with resulting enhanced depth of focus. Nevertheless, the data show that, in 28.9% of the evaluated eyes, BCVA was reduced after implantation of the artificial iris. Therefore, patients should be advised prior to surgery that artificial iris implantation has a potential risk to reduce the patients' vision due to the procedure itself.

The local postoperative therapy with steroids and antibiotics is similar to the therapy in other intraocular procedures. In principle, there is no contraindication for a use of dilating eyedrops in case of intraocular inflammation, although there is no effect on the fixed pupil diameter. However, it may help to reduce the inflammatory reaction due to inhibited ciliary body motility. Another positive effect when dilating the pupil is the fact that there is potentially less pigment dissolving from the residual iris and consecutively a lower risk for secondary pigmentary glaucoma.

In this case with moderate recurrent bleeding into the anterior chamber and secondary rise of the intraocular pressure, the used artificial iris with embedded fiber meshwork was trephanized and then sutured in the ciliary sulcus. The ultrasound and clinical examination did not reveal any detectable bleeding source. The bleeding stopped spontaneously after 3 months with the need for topical and systemic antiglaucomatous medication and topical cycloplegics for this period of time.

**Figure 8:**
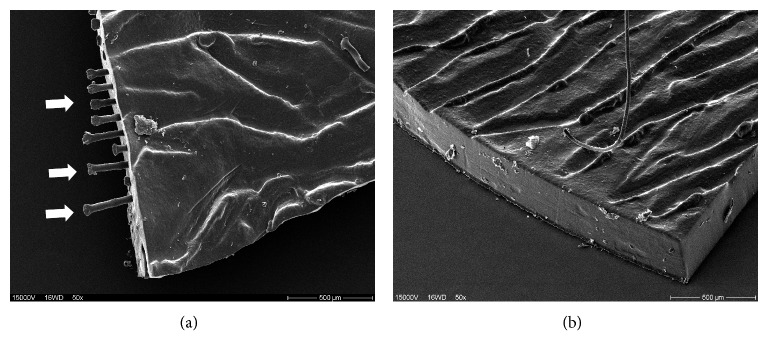
Electron microscopic images of the edge of an artificial iris with embedded fiber meshwork (a). Outstanding sharp-edged fibers of the smooth silicon tissue are shown (arrows) caused eventually by use of a reusable trephine in this case. These could cause chronic bleeding in contact areas between the artificial iris and adjacent intraocular surfaces. (b) Smoothly trephined edge of an implant without embedded fiber meshwork with a single-use trephine.

Scanning electron microscopy of the leftover parts of the trephanized iris showed a variety regarding the smoothness and roughness of the edges and the protrusion of fibers in implants with fiber (Figure 8(a)) and without fiber meshwork (Figure 8(b)), eventually depending on the sharpness of the used trephines.

It seems that chronic movement of the trephined artificial iris edges on the ciliary body leading to defects on the ciliary body may have been the reason of the observed recurrent bleedings. As a consequence, from that incidence on, we have always used single-use sharp trephines to achieve clean cutting edges in view of eliminating this possible problem.

Early transient postoperative IOP rise is normally well controlled with antiglaucomatous eyedrops and systemic carboanhydrase inhibitors. The one case with prolonged and severe IOP rise due to pigment dispersion syndrome that was not sufficiently controlled with antiglaucomatous medication required glaucoma shunt surgery. This mentioned patient had a dark brown residual iris, implicating eventually a higher risk for pigmented dispersion in patients with dark irides. We were not able to differentiate the cause for IOP decompensation in the other patients (decompensated glaucoma versus artificial iris implantation).

On the other side of the spectrum, temporary bulbar hypotonia usually depended on the surgical technique especially in case of larger entries for specific lens diameters and sutureless pars plana vitrectomies. The expected hypotonia in those cases resolved within one week and was not a problem at any time [23].

A challenging situation may occur when a sector-shaped artificial iris is implanted, for example, in cases with partial aniridia or coloboma (Figure 1(a)): Constant movement of the residual iris can result in cutting sutures especially at the rim of the pupillary sphincter (Figure 1(b), arrow) with a secondary pupil and disturbed vision. This happened to both sector-shaped implants in our study. Because of that and the fact that the implantation procedure is more complex because of time-consuming iris sutures, we, since then, avoided this implantation technique with sector-shaped artificial iris implants [24]. The advantage to use a complete artificial iris over a sector-shaped one is the easier implantation procedure, better predictable centration of a perfectly round pupil, avoiding of sharp implant edges and prevention of cutting sutures. Primary sutures for the artificial iris fixation are recommended and necessary in cases with missing anterior and/or posterior device support, for example, in aphakic eyes or absent of the lens capsules [24].

Suture-fixed implants last as long as the material of the sutures endures. In this series, we used durable polypropylene 10-0 sutures (Ethicon®). This UV-stable material should last at least for several years [25]. The postoperative follow-up time for our first operated patients is 5 years. Future follow-up examinations will show whether this fixation will last in younger patients with a life expectancy longer than the life span of the suture material. Supposedly, similar to suture-fixed ciliary sulcus IOLs, the sutured iris implants should hold less potential for complications.

Subluxations occurred either within the first two days or only after months after the implantation procedure (Figures 2 and 3). Decentration of the new pupil may result in difficulties to choose the correct diameter of the implant as well as presumed mechanical manipulation by the patient. Furthermore, loosening of the sutures (e.g., caused by aging) can cause a dislocation. The reasons for the early subluxations were preexisting scars, synechiae, adhesions at the implantation site, or loose structures resulting from the former trauma. This may lead to unstable artificial iris fixation. Exemplary, postoperative eye movement or gravity can force the artificial iris to leave its intended position, forming a “slide-off phenomenon” (Figure 3): The artificial iris tilts to one side of the suture axis heading towards the vitreous with the risk of causing severe mechanical retinal damage. In artificial irides without embedded meshwork, sutures could cut through the silicone tissue more easily. Those sutures prevent the refixated artificial iris from swinging on the suture-fixed intraocular lens and hold it in place (Figure 3(f)). Removing the artificial iris out of the posterior segment would need a removal of both, the artificial iris and the intraocular lens, to be removed and then refixated. Until now, we could not observe a suture cut through in fiber-free implants.

In our group, we had one case with retinal detachment that had to undergo vitreoretinal surgery 4 weeks after implantation of the artificial iris. Vitreoretinal surgery in general is feasible because of the fixed pupil size of the implanted artificial iris. The fundus of the affected eyes can be examined up to the periphery despite the lack of pupil dilation. This can be seen during implantation surgery of an artificial iris. The visualization extends up to the peripheral retina, even the ora could be visualized using indentation.

As described in a previously published work, the corneal endothelium cell loss is about 11.3% and comparable to a standard cataract surgery [23]. The amount of endothelial cell loss caused by the implantation of an artificial iris itself is not significantly higher than after standard cataract surgery, but the preoperative circumstances after severe ocular trauma often result in a reduced endothelial cell count before surgery. Increased loss of endothelial cells is an additional risk factor of the patients' traumatized eyes and remains the difficulty in the reconstruction surgery. In anticipation of that, three patients needed keratoplasty after artificial iris implantation. Therefore, the risk for corneal decompensation is more likely to be attributed to the primary ocular circumstances than the surgical trauma. Regarding the patient with corneal decompensation after artificial iris implantation and macular edema, we successfully removed the artificial iris implant and performed a keratoplasty that had no complications during and after surgery.

## 5. Conclusion

In conclusion, iris reconstruction with ArtificialIris® implantation is an outstanding and rare ophthalmic surgery with potential unexpected events and a profoundly improving learning curve after a dozen implantations. After artificial iris implantation, the postoperative development can reveal unexpected adverse events of various quality and quantity. In summary, pupillary reconstruction with the used artificial iris implant should not be underestimated regarding its follow-up management and therefore requires significant surgical experience to avoid unnecessary pitfalls. Consequently, we would advise the use of artificial iris implants in specialized centers and focus on few surgeons.

## Figures and Tables

**Table 1:** Patients' characteristics and descriptive data.

Number of subjects	*n*=51
Age (years)	52.9 ± 16.0
Gender	
Male	22 (68.8%)
Female	10 (31.2%)
Iris defects resulting from	
Congenital coloboma	3 (9.3%)
Persistent mydriasis	9 (28.1%)
Traumatic loss of iris tissue	21 (65.6%)
Additional history information	
Preexisting glaucoma	6 (11.8%)
Preexisting corneal impairment (scars or decompensation)	5 (9.8%)
Time period between diagnosis and surgery (years)	12.0 ± 15.7
Follow-up time (months)	13.6 ± 10.9

**Table 2:** Graduation of complications related to the artificial iris (AI) implantation.

Grade	Description	Event	*n*	% of total *n*=51
0 (none)	Postoperative uneventful course of disease		38	74.5
1 (mild)	Unexpected events needed for noninvasive intervention *with* full recovery	Recurrent bleedings with IOP raise	1	2.0
		Minor but stable deviation of the iris	1	2.0
		Need for laser-assisted capsulotomy	2	3.9
2 (moderate)	Unexpected events needed for noninvasive intervention *without* full recovery	Suture cut through residual iris	1	2.0
		New onset of glaucoma with need for local therapy	3	5.9
		New onset of clinically corneal decompensation	3	5.9
3 (severe)	Unexpected or expected events leading to any surgical intervention	Suture loosening of AI/lens	2	3.9
		Artificial iris sub-/dislocation	3	5.9
		New vitreous strands or synechiae in the anterior chamber	2	3.9
		New onset or aggravation of glaucoma with need for surgical intervention (valve drainage device)	2	3.9
		New onset or aggravation of clinically corneal decompensation with a need for amniotic membrane transplantation	2	3.9
		New onset or aggravation of clinically corneal decompensation with a need for keratoplasty	5	9.8
		CME	3	5.9
		Retinal detachment	1	2.0

## Data Availability

The data used to support the findings of this study are available from the corresponding author upon request.

## Supplementary Materials

Supplementary Materials Video: off-center artificial iris revise of the patient in Figure 2(e): the subluxated artificial iris device is held on to it firmly before vanishing into the posterior segment. The prosthesis is repositioned and prepared for suture fixation by threading the margin of the implant and sutured to the sclera.
